# Mores of the customer base for ecotourism industry: Development and validation of a new measurement scale

**DOI:** 10.1371/journal.pone.0246410

**Published:** 2021-02-18

**Authors:** Shahid Bashir, Muddasar Ghani Khwaja, Asif Mahmood

**Affiliations:** 1 Department of Business Studies, Namal Institute, Mianwali, Pakistan; 2 Management Sciences, Shaheed Zulfikar Ali Bhutto Institute of Science and Technology, Islamabad, Pakistan; University of Naples Federico II, ITALY

## Abstract

To date, there is no such scale that may precisely measure mores of the customer base for the ecotourism industry. Therefore, a thematic analysis of literature has been conducted by examining various good quality research works on intrinsic characteristics eliciting pro-environmental actions. Based upon the thematic analysis, a new scale of measure has been proposed with the help of 17 scholars and 15 practitioners hailing from different countries by mutually agreed intended meanings and breadth of the theoretical concepts. The new scale has 4 dimensions comprising a pool of 32 items, which has been empirically validated through the data collected from 268 Malaysian tourists. The dimensions are: sense of obligation to care for the natural environment, sense of obligation to practice eco-friendly activities, sense of obligation to purchase eco-friendly products, and sense of obligation to support eco-friendly inventions. The theoretical and managerial implications together with research limitations have been discussed.

## Introduction

The acquired intrinsic characteristics due to widely observed norms within a particular society are commonly referred to as ‘mores’ [1]. As the interest of the new generation in tourism is increasing, the buying decisions related to ‘mores’ have become often the principal source behind the development of ecotourism trends. Ecotourism readiness is growing at a rapid pace with the growing mores among the young tourists related to sustainable and ethical purchase decisions [2]. [3] report that around 35% of tourists confirmed ecotourism during the fourth quarter of the year 2016. World Tourism Organization expects the ecotourism market to climb up to $1.8 billion during the year 2030 [4].

The tourism business model includes various components such as accommodation, transportation, food and recreation [5]. When the concept of eco is added to the tourism business model, the element of environment conservation and well-being is also considered specifically. For instance, eco-accommodation companies (or hotels) facilitate their customers with conserved energy, limited water waste, ditched disposals, reusable linens and cleaned supplies [6–8]. Similarly, eco-transport companies provide their customers with electric and cell-powered vehicles (e.g., train, planes and cars) and compressed aired, Stirling-powered and fine-tuned conventional cars [7,9–16]. Likewise, eco-food companies (or restaurants) serve their customers with local and organic food, composite of food waste, eco-friendly food packaging and filtered water dispensers [16–21]. In the same way, eco-recreation companies (or local trip agents) facilitate their customers with travels toward those places where flora and wildlife are the primary attractions, and usually involve visiting fragile, pristine and relatively undisturbed natural areas [6–8].

The practitioners, who are currently employing or planning to make use of ecotourism business models, ascribe this phenomenon to a real revolution. For example, the managing director of Catalonia’s Tourism Agency, David Font, states (in [22]) that tourism without sustainability does not have any future. Even while the current market size of ecotourism is 2.7% low compared with the conventional tour business, the profit margin is around 7% high [22]. Therefore, a growing number of SMEs and entrepreneurs are getting engaged in this sector with their customized environment-friendly service offers.

In order to make more profitable and attractive recognition in the tourism industry, the existing and future business models of ecotourism must precisely understand the mores of the customer base (or tourists) [23]. For that reason, an insight can be taken from academic researches (such as of [11,24–26]). For instance, while developing an extended model of the theory of planned behavior through predicting intentions of consumers toward green hotel visits, [11] observed that environmentally concerned individuals usually oblige themselves morally toward making positive intentions during their visits to the eco-friendly hotel. In their study, the evidence suggested that perceived moral obligation was the lowest predictor of intention to visit green hotels. This would suggest that moral obligation is only a marginal drive compared to the other more rational antecedents (attitude, subjective norm, PBC).

[24] added to that concept while arguing that individuals intend toward visiting eco-friendly hotels when they feel morally obliged as a result of their perceived biosphere (layer of earth where life exists) values, awareness of consequences and ascription of responsibility for global warming, energy and fuel issues. Their study could raise an argument over research findings in line with the Value-Belief-Norm chain, since they did not test any mediation from biosphere value to intention via awareness of consequences and ascription of responsibility.

Moreover, while analyzing the environmental concerns and consumer behavior about food packaging, [25] noticed that moral norms could lead to positive buying and recycling behavior. Furthermore, while investigating the impetus of a traveler’s willingness to go green, [26] observed that the sense of obligation to take pro-environmental actions could eventually lead toward usage and recommendation of electric airplanes. However, these researchers actually found that the association between moral norms and pro-environmental consumption intention was not significant.

In sum, illustrating the mixed results in the literature would strongly support the necessity of measuring mores of customer base toward pro-environmentalism in more detail. Nevertheless, as the ecotourism trend is rapidly expanding [4], and the research work on intrinsic characteristics has addressed issues about free will and determinism of the individuals [27], understanding tourists’ mores has become difficult. Consequently, a particular statement or proposition, despite sound reasoning from the scholars, could lead toward a conclusion that seems logically unacceptable or self-contradictory for a practitioner [28]. For example, if a person feels morally obliged to travel through an environmentally sound mode of transportation, “will he do the same even when it is more expensive or time-consuming?”, or “will he do the same regardless of what other people view about him”, or “will he feel guilty if he is unable to travel through the environmentally sound mode of transportation”, or “will he feel that traveling through an environmentally sound mode of transportation makes him a better person” etc.

Besides, the operation of neoliberal business and management process has revived and partially reinvented some traditional mores for their customers [29]. Some conservation entrepreneurs, however, want to go further looking to explicitly and intentionally develop a business model and conservation tourist project that does zero percent ecological harm and also ensures that local communities are genuine beneficiaries [29]. To support such conservation entrepreneurs, plus to more precisely understand tourists’ mores, a suitable scale is required that can measure mores of the customer base for the ecotourism industry.

To date, no such scale exists, even though the concept of social norms has been often studied (e.g., by [23,30]). Keeping in view the mentioned grounds, the goal of this study is, firstly, to conduct a thematic analysis of the existing literature by examining various good quality research works on the intrinsic characteristic that elicits pro-environmental actions, and secondly, based upon the thematic analysis, to propose a new scale that can measure mores of the customer base for the ecotourism industry.

The present study makes several theoretical and practical implications by advancing contemporary literature and adding value to the ecotourism industry. It develops mutually agreed intended meanings and breadth of the theoretical concepts, with the help of 17 scholars and 15 practitioners hailing from Asia, Europe and America, by adopting a rigorous procedure of scale development, as suggested by [31]. Afterward, it refines and validates the newly established scale through survey data collected from the Malaysian tourists to provide a more worldwide view to the existing literature, as most of the existing literature in a similar context comes from the Western countries. Through this study, a better understanding of the mores of the customer base will primarily support practitioners to gain high ecotourism market share soon. Besides, since this study emphasizes toward a holistic approach to achieving sustainable development, it would help accomplish two of the Sustainable Development Goals (SDGs) adopted by the General Assembly of the United Nations in their Agenda 2030 [32], namely, Sustainable Cities and Communities, and Responsible Consumption and Production.

The subsequent section includes a thematic analysis of literature intrinsic characteristic that elicits pro-environmental actions. Later on, the research methodology will be presented, which includes intended meanings and breadth of the theoretical concepts, survey, and scale refinement. Afterward, the discussion and conclusion will be presented to bring the overall research into perspective.

### Thematic analysis of literature

[33] describe that people oblige themselves to perform moral behavior upon facing an ethical situation. In a pro-environment behavioral context, such obligations are referred to as a basis for the people’s transition from their general predispositions to pro-environmental actions [34]. Contrarily, the less an individual is aware of human actions that can cause threats to the environment, the less (s)he will feel morally obliged to perform pro-environmental behavior [35,36]. Several scholars (i.e., [37–39]) argue that people usually perceive other’s anti-environmental actions based on their intrinsic characteristics (the character individuals have for themselves, independent from others, and including their own context), and then motivate themselves to oblige toward performing pro-environmental actions. Accordingly, identification of an individuals’ intrinsic characteristics (or factors) is of utmost critical to explain their pro-environmental actions [38–40]. Therefore, in this study, various tourism-related scholarly contributions have been analyzed to gain an insight into those intrinsic characteristics which can motivate an individual to oblige toward performing certain pro-environmental actions as collated in Table 1.

**Table 1:** Intrinsic characteristic eliciting pro-environmental actions.

Intrinsic characteristic	Pro-environmental actions	References
Sense of obligation to care for the natural environment	• Save and protect natural resources (i.e., air, water, and land), • Reduce their impacts on global warming and climate change, and • Develop an eco-friendly behavior.	[6,8,39,41,42]
Sense of obligation to practice eco-friendly activities	• Traveling through environmentally sound modes of transportations (e.g., electric vehicles, hydrogen-fueled vehicles, compressed aired vehicles, Stirling-powered vehicles, and fine-tuned vehicles), • Practicing eco-friendly accommodation (or hotel) and recreation, • Reducing electricity usage, and • Increasing eco-friendly packaging usage.	[6–16,21]
Sense of obligation to purchase eco-friendly products	• Purchasing recycled products, • Purchasing organic food products (e.g., fresh meat and dairy, processed foods, and frozen meals), • Purchasing organic clothing products (e.g., outfit composed of cotton, silk, jute, wool, or ramie), and • Purchasing organic personal care products (e.g., made of plant or derived from naturally occurring ingredients).	[16–20,43,44]
Sense of obligation to support eco-friendly inventions	• Supporting all those inventions (or innovations) which are less harmful to the environment (e.g., the electric airplane).	[10,39,45]

The first intrinsic characteristic is ‘sense of obligation to care for the natural environment’. Due to this, people oblige themselves to save and protect natural resources (i.e., air, water and land), accepting the fact that they are limited [6,8,39]. Moreover, this intrinsic characteristic becomes people’s source of motivation to reduce their impacts on global warming and climate change, and expect such demeanor even from their future generations [41,42]. Furthermore, this intrinsic characteristic motivates people to develop eco-friendly behavior regardless of what others think about them [6,8].

The second intrinsic characteristic is ‘sense of obligation to practice eco-friendly activities’. This trait motivates an individual to oblige toward traveling through environmentally sound modes of transportations (e.g., electric vehicles, hydrogen-fueled vehicles, compressed aired vehicles, Stirling-powered vehicles and fine-tuned vehicles) [9–16], even if it takes extra time and/or is more expensive [7]. Moreover, this intrinsic characteristic motivates an individual to oblige toward practicing eco-friendly accommodation (or hotel) and recreation [6–8]; reducing electricity consumption [6,8]; and increasing eco-friendly packaging usage [21].

The third intrinsic characteristic is ‘sense of obligation to purchase eco-friendly products’. Due to this, people oblige themselves to purchase recyclable and organic products [16–20]. As an eco-friendly approach for organic products, the studies by [16,18,19] have considered only organic food products which are grown under an agriculture system without using harmful pesticides and chemical fertilizers. However, several scholars (i.e., [43,44]) expressed that organic products include clothing and personal care products as well. Organic clothing refers to products prepared using such raw materials which have been produced using organic methods. Whereas, organic personal care products refer to the items prepared using agricultural ingredients without chemical inputs. Perhaps, both categories (organic clothing and organic personal care products) could motivate an individual to oblige toward purchasing eco-friendly products. Hence, both have been considered for this study.

The fourth intrinsic characteristic is ‘sense of obligation to support eco-friendly inventions’. Within the current literature, there is not much attention laid out related to this intrinsic characteristic. However, few scholarly contributions (i.e., [10,26,45]) were found intuitive. In the most recent study on this intrinsic characteristic, [26] noted that there exists a motivation among people to oblige toward traveling through the electric airplane (less harmful to the environment), which is a likely invention soon. Consequently, their study can be used as a unique insight.

## Research methodology

**Fig 1:**
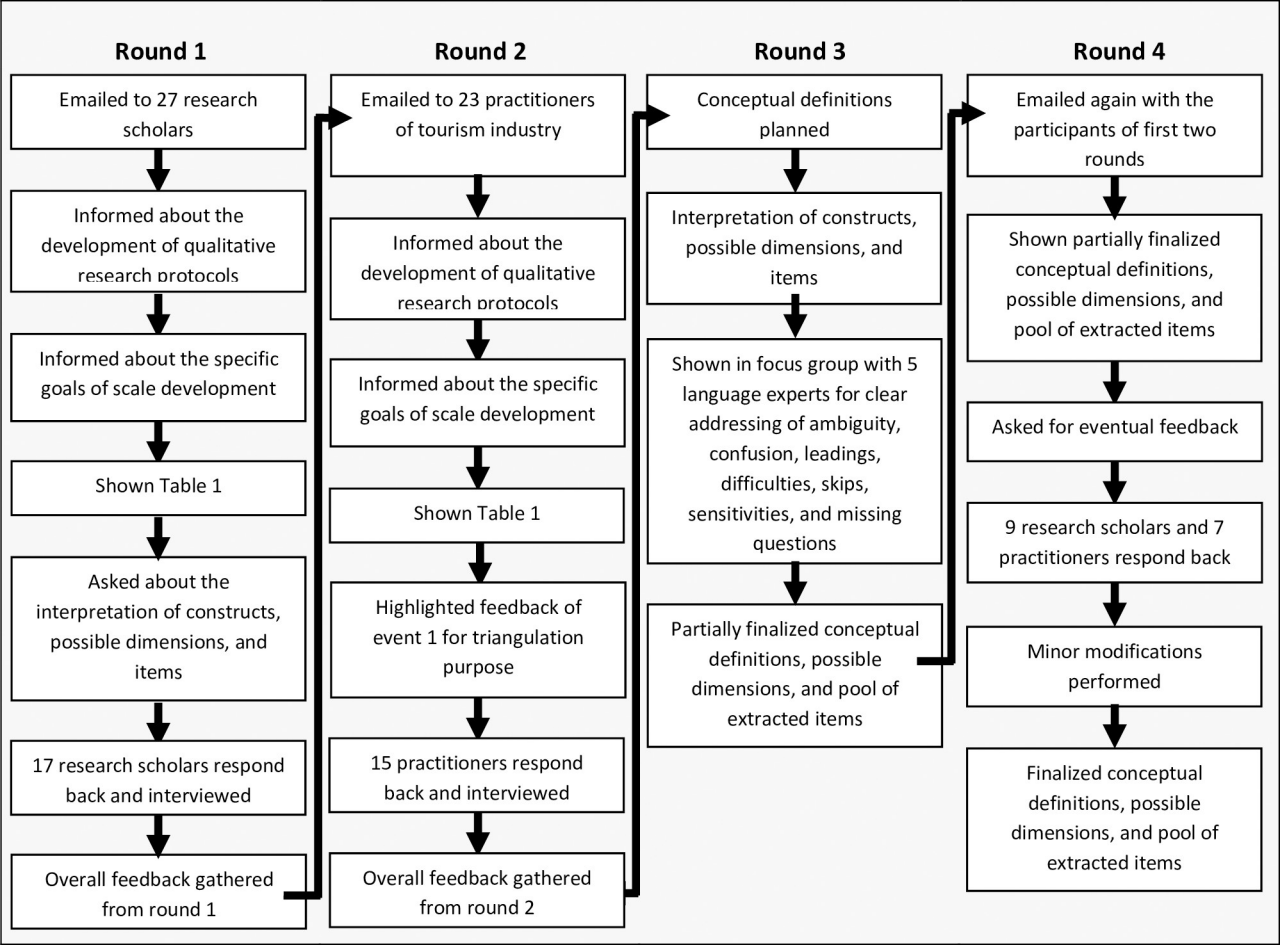
The procedural steps adopted to develop intended meanings and breadth of the theoretical concepts.

Based on the thematic analysis, scholars and practitioners were involved in this study for the development of mutually agreed intended meanings and breadth of the theoretical concepts. This rigorous procedure has been recommended by various research scholars (i.e., [31,46–51]) for proper and efficient theory development. Accordingly, several rounds of information gathering, as suggested by [31], were conducted. The summary of the procedural steps adopted during these rounds is presented in Fig 1.

### Ethics statement

Ethical review and approval were not required for this study on human participants in accordance with the local legislation and institutional requirements. Moreover, the participants provided their written informed consent to participate in this study.

### Intended meanings and breadth of the theoretical concepts

As seen in Fig 1, the information was gathered in a total of 4 rounds. In the first and second rounds, participants were the scholars and practitioners respectively. The scholars were from Asia, Europe and America whose area of interest was related to tourism and hospitality, and their research work in a similar context has been published in well-reputed journals. Whereas, the practitioners were from South-East Asian countries where ecotourism is actively taking place [52]. They had at least five years of work experience as part of senior management teams of eminent tourism companies along with at least two years of experience to organize eco-friendly tours.

Through these two rounds, the information was gathered from a total of 32 participants, which is a good number for the most comprehensive assessments [53]. Based on the information gathered, the conceptual definitions were developed along with the interpretation of constructs, possible dimensions and items. The combined tersely outcomes from the first and second rounds were:

- Almost all the participating academicians and practitioners agreed that the idea of exploring mores of the customer base in a deeper manner is novel considering the current scenario of the global tourism industry.
- Nearly all of them agreed that four intrinsic characteristics such as the sense of obligation to care for the natural environment, sense of obligation to practice eco-friendly activities, sense of obligation to purchase eco-friendly products, and sense of obligation to support eco-friendly invention can act to motivate an individual to oblige toward performing certain pro-environmental actions as shown in Table 1.
- Just about three-fourths of them (mostly academicians) were concerned that the items chosen to represent mores of the customer base may not apply to the overarching construct in consistent ways. Accordingly, as mentioned in Table 1, the intrinsic characteristics should be considered as possible dimensions, whereas the pro-environmental actions as possible items to measure overall mores of the customer base.
- Five of them suggested, during the evaluation of possible dimensions and items, that the moral obligation of individuals should be probed further while knowing their level of guilt toward environmental harm, as well as the valuable contribution made toward society. Their precise suggestions in this regard were noted to take the overall discussion in perspective.

Consequently, based on the information gathered in the first two rounds, the overarching construct (i.e., mores of the customer base) was proposed to be split into four different dimensions, with a pool of 36 items. According to [31], vagueness in responses, biased arguments, and measurement errors can arise due to the use of complex words or language. As a result, in the third round, a focus group was conducted among five language experts. During the focus group sessions, the participants were first shown the outcomes of the first two rounds (i.e. conceptual definitions, possible dimensions, and the pool of extracted items). Second, they were asked questions such as ‘Is this definition easy to understand?’ ‘What does this concept mean to you?’ ‘Are the items shown under a particular dimension the same?’, ‘What do you think while reading this particular question?’ etc. Third, their behavioral coding was also observed while watching whether they frowned or hesitated once they were asked to read any question. Altogether, their suggestions were found to be useful because several areas were addressed, such as ambiguity, confusion, leadings, difficulties, skips, sensitivities and missing questions. For instance, they suggested that various terms and concepts have to be explained with suitable examples for better understanding of the respondents, such as natural resources, environmentally sound modes of transportations, eco-friendly accommodation, and organic products. Hence, their suggestions were noted down to take the overall discussion in perspective.

In the fourth round, the information compiled through the first three rounds was emailed again to the participants of the first two rounds. The goal of this round was to receive eventual feedback on the quality of definitions, dimensions and items. Consequently, a new 4-dimensional overarching construct, named as *Mores of the Customer Base*, was conceptually defined as ***the sense of obligation to care for the natural environment*, *practice eco-friendly activities*, *purchase eco-friendly products*, *and support eco-friendly inventions*, *which can motivate an individual to oblige toward performing certain pro-environmental actions*.** Similarly, the dimensions of the construct have also been spelled out, and presented in Table 2.

**Table 2:** The possible dimensions and pool of extracted items.

Possible Dimension	Conceptual Definition	Proposed Items
Sense of Obligation to Care for Natural Environment (SOCNE)	An intrinsic characteristic which can motivate an individual to oblige toward saving and protecting natural resources, reducing the impacts on global warming and climate change, and/or developing an eco-friendly behavior, so that the guilt of environmental harm is reduced and/or a valuable contribution to the society is made.	• I feel morally obliged to save and protect natural resources (i.e., air, water, and land) (SOCNE 1). • I feel guilty if I am unable to save and protect natural resources (SOCNE 2). • Saving and protecting natural resources feels me a better person (SOCNE 3). • I feel morally obliged to reduce the impacts on global warming and climate change (SOCNE 4). • I feel guilty if I am unable to reduce the impacts on global warming and climate change (SOCNE 5). • Reducing the impacts on global warming and climate change feels me a better person (SOCNE 6). • I feel morally obliged to develop eco-friendly behavior (SOCNE 7). • I feel guilty if I am unable to develop eco-friendly behavior (SOCNE 8). • Developing an eco-friendly behavior feels me a better person (SOCNE 9).
Sense of Obligation to Practice Eco-friendly Activities (SOPEA)	An intrinsic characteristic which can motivate an individual to oblige toward traveling through the environmentally sound mode of transportation, practicing eco-friendly accommodation and recreation, reducing electricity usage, and/or using eco-friendly packaging so that the guilt of environmental harm is reduced and/or a valuable contribution to the society is made.	• I feel morally obliged to travel through the environmentally sound mode of transportation (e.g., electric vehicles, hydrogen-fueled vehicles, compressed aired vehicles, Stirling-powered vehicles, and fine-tuned vehicles) (SOPEA 1). • I feel guilty if I am unable to travel through the environmentally sound mode of transportation (SOPEA 2). • Traveling through the environmentally sound mode of transportation feels me a better person (SOPEA 3). • I feel morally obliged to practice eco-friendly accommodation (e.g., hotel) and recreation (e.g., fun, visits, camping) (SOPEA 4). • I feel guilty if I am unable to practice eco-friendly accommodation and recreation (SOPEA 5). • Practicing eco-friendly accommodation and recreation feels me a better person (SOPEA 6). • I feel morally obliged to reduce electricity usage (SOPEA 7). • I feel guilty if I am unable to reduce electricity and water usage (SOPEA 8). • Reducing electricity and water usage feels me a better person (SOPEA 9). • I feel morally obliged to use eco-friendly packaging (SOPEA 10). • I feel guilty if I am unable to use eco-friendly packaging (SOPEA 11). • Using eco-friendly packaging feels me a better person (SOPEA 12).
Sense of Obligation to Purchase Eco-friendly Products (SOPEP)	An intrinsic characteristic that can motivate an individual to oblige toward purchasing recycled and/or organic products so that the guilt of environmental harm is reduced and/or a valuable contribution to the society is made.	• I feel morally obliged to purchase recycled products (SOPEP 1). • I feel guilty if I am unable to purchase recycled products (SOPEP 2). • Purchasing recycled products feels me a better person (SOPEP 3). • I feel morally obliged to purchase organic food products (e.g., fresh meat and dairy, processed foods, and frozen meals) (SOPEP 4). • I feel guilty if I am unable to purchase organic food products (SOPEP 5). • Purchasing organic food products feels me a better person (SOPEP 6). • I feel morally obliged to purchase organic clothing products (e.g., outfit composed of cotton, silk, jute, wool, or ramie) (SOPEP 7). • I feel guilty if I am unable to purchase organic clothing products (SOPEP 8). • Purchasing organic clothing products feels me a better person (SOPEP 9). • I feel morally obliged to purchase organic personal care products (e.g., made of plant-derived and naturally occurring ingredients) (SOPEP 10). • I feel guilty if I am unable to purchase organic personal care products (SOPEP 11). • Purchasing organic personal care products feels me a better person (SOPEP 12).
Sense of Obligation to Support Eco-friendly Inventions (SOSEI)	An intrinsic characteristic that can motivate an individual to express their future obligations toward supporting all those inventions (or innovations) which are less harmful to the environment, so that the guilt of environmental harm is reduced and/or a valuable contribution to the society is made.	• I will feel a moral obligation to support all those inventions (or innovations) which are less harmful to the environment (SOSEI 1). • I will feel guilty if I am unable to support all those inventions (or innovations) which are less harmful to the environment (SOSEI 2). • I will be a better person by supporting all those inventions (or innovations) which are less harmful to the environment (SOSEI 3).

### Survey

In order to validate the new 4-dimensional overarching construct, the Malaysian tourists were selected as the participants for a 5-point Likert scaled (strongly disagree to strongly agree) administrative survey data collection. The country, located in South-East Asia, is well known for its lively participation in ecotourism. Several chains of hotels in Malaysia (such as Mandarin Oriental Hotel, Shangri-La Hotel and Renaissance Kuala Lumpur) have won the title of ASEAN Green Hotels [54]. Therefore, examining the viewpoints of the Malaysian tourists can be extremely significant in terms of presenting a more world-wide insight into the existing literature.

As part of the initial screening, it was ensured that respondents must have heard about the ecotourism, and have ever had a direct experience of purchasing an ecotourism package. If they had purchased an ecotourism package more than once in the past year, they had to relate their responses based on the overall experience. Before launching the survey, a test-run was performed on 45 tourists in Malaysia. That test-run was evaluated using Cronbach’s alpha test, which reached 0.892, meaning that the survey was set for a full-scaled administration.

The survey was conducted using the services of three well-reputed panel companies (firms that match online respondents with the target audience of the survey for a fee per complete response) in Kuala Lumpur. The survey was accompanied by a cover letter with an explanation of the research objectives, as well as the assurance of the confidentiality of the participant’s responses. That practice turned out to be useful, as the overall response rate was 64%. Moreover, the age and gender profile of the respondents were found to be consistent with the official portal of the [55]. Furthermore, the personal income profile of the respondents was also observed to be nearly in line with the Statista Global Consumer Survey-2019 [56].

The total number of respondents for this study was 268 (an acceptable sample size as suggested by [49,57–59]). Their demographic results demonstrate that all the respondents (100%) heard about ecotourism as shown in Table 3. The majority of the respondents (52.3%) were females, while male respondents were 47.7%. A large number of respondents fell in the age category of below 30 years (40.6%), while people having the age of 50 years or above were only 25 (9.6%). Many of the respondents had undergraduate degrees 148 (55.3%), while graduate respondents were 85 (31.9%), and people with other qualifications were 35 (12.8%). The income level of the respondents revealed that the number of respondents having earnings $7,000 –$11,500 (per annum) was 141 (52.5%), respondents with income below $7,000 were 65 (24.2%), whereas respondents with earnings more than $11,500 were 62 (23.3%).

**Table 3:** Demographic profile (N = 268).

Items		Frequency	Percentage
Gender	Male	128	47.7%
	Female	140	52.3%
Age (in years)	Below 30	109	40.6%
	31–40	82	30.5%
	41–50	52	19.3%
	51–60	14	5.2%
	Above 60	11	4.4%
Education	Undergraduates	148	55.3%
	Graduates	85	31.9%
	Others	35	12.8%
Income Level	Below $7,000	65	24.2%
	$7,000 –$11,500	141	52.5%
	Above $11,500	62	23.3%
Frequency of purchasing	One time	26	9.6%
ecotourism package during	Two times	34	12.6%
last year	Three times	90	33.8%
	Above three times	118	44.0%
Heard about green hotels	Yes	268	100%
	No	0	0

### Scale refinement

Before proceeding with the scale refinement and validation procedure, testing for outliers, linearity and multicollinearity was performed using SPSS 22 (recommended by [60]). The overall results confirmed clean data with only a few outlier cases. Their patterns were inspected, and modified to avoid impact on the overall outcomes. Besides, to examine normality related issues in the data, the statistics of skewness and kurtosis were computed using SPSS 22. For all sub-constructs, the standard deviation values were below 1, the skewness values were between -2 and 2, and the kurtosis values were between -3 and 3, which demonstrate normality [61,62].

Then to proceed with factor analysis, the values of Kaiser-Meyer-Olkin (KMO) (min. value>0.6) and Bartlett’s test of sphericity (<0.05) were observed [63,64], and were found to meet the criteria. Afterward, exploratory factor analysis (EFA), using the maximum likelihood estimation method with oblique rotation (Eigenvalue >1), was conducted on the data to ensure loadings of the items on their respective factors. The outcome of EFA, based upon the standards of non-fixed factor extraction (KMO > 0.6 and Bartlett’s test of sphericity < 0.05), disclosed that the following items *SOPEP 5*, *SOPEA 11*, *SOCNE 5* and *SOCNE 8* did not load on their respective factors. Thus, after removing these items, EFA results provided a pattern matrix without any cross-loading or missing loading as shown in Table 4.

**Table 4:** Pattern Matrix- Exploratory Factor Analysis (EFA).

	1	2	3	4
SOPEP1	0.531			
SOPEP2	0.534			
SOPEP3	0.747			
SOPEP4	0.602			
SOPEP6	0.851			
SOPEP7	0.819			
SOPEP8	0.812			
SOPEP9	0.580			
SOPEP10	0.782			
SOPEP11	0.666			
SOPEP12	0.734			
SOPEA1		0.638		
SOPEA2		0.833		
SOPEA3		0.779		
SOPEA4		0.793		
SOPEA5		0.843		
SOPEA6		0.905		
SOPEA7		0.603		
SOPEA8		0.420		
SOPEA9		0.443		
SOPEA10		0.401		
SOPEA12		0.457		
SOCNE1			0.765	
SOCNE2			0.736	
SOCNE3			0.793	
SOCNE4			0.929	
SOCNE6			0.934	
SOCNE7			0.743	
SOCNE9			0.768	
SOSEI1				0.821
SOSEI2				0.813
SOSEI3				0.815

To ensure no common method biases in the collected data, a comparison was made between the 4-dimensional model and one-factor model that treated 36 items as a common factor. The outcomes of the one-factor model confirmed a poor model fit, as the majority of communalities (<0.50) and factor loadings (<0.40) were low. Furthermore, in order to check the possibility of the construct modeling on a more abstract higher-level dimension, a second-order model (e.g., guilt + moral obligation could be rational and emotional dimensions of each considered "sense of obligation") was tested. However, their results turned to be a poor model fit, because the majority of communalities (<0.50) and factor loadings (<0.40) were low, and the loadings of items on their respective factors could not be established. This means that a 4-dimensional model is a better fit and there is no common method bias in the outcomes.

Consequently, the affirmative outcomes attained from EFA (Table 4) motivated us to conduct confirmatory factor analysis (CFA) to further endure the strength and association of items with the respective constructs. The depiction of CFA can be seen in S1 Fig, whereas detailed statistical outcomes of CFA have been provided in Table 5.

**Table 5:** Factor loadings of confirmatory factory analysis (N = 268).

Constructs & Items	Standardized factors loadings using CFA
***Sense of Obligation to Care for Natural Environment***	
SOCNE 1	0.845
SOCNE 2	0.867
SOCNE 3	0.850
SOCNE 4	0.851
SOCNE 6	0.802
SOCNE 7	0.741
SOCNE 9	0.629
***Sense of Obligation to Purchase Eco-friendly Products***	
SOPEP 1	0.605
SOPEP 2	0.594
SOPEP 3	0.734
SOPEP 4	0.624
SOPEP 6	0.842
SOPEP 7	0.762
SOPEP 8	0.814
SOPEP 9	0.681
SOPEP 10	0.764
SOPEP 11	0.554
SOPEP 12	0.755
***Sense of Obligation to Practice Eco-friendly Activities***	
SOPEA 1	0.633
SOPEA 2	0.819
SOPEA 3	0.747
SOPEA 4	0.780
SOPEA 5	0.865
SOPEA 6	0.898
SOPEA 7	0.690
SOPEA 8	0.466
SOPEA 9	0.484
SOPEA 10	0.459
SOPEA 12	0.465
***Sense of Obligation to Support Eco-friendly Inventions***	
SOSEI 1	0.881
SOSEI 2	0.883
SOSEI 3	0.861

χ2 = 924.494, df = 449, χ2/df = 2.059, P = 0.000, GFI = 0.820, AGFI = 0.788, RMSEA = 0.063, SRMR = 0.061, CFI = 0.919, NFI = 0.856 and TLI = 0.911.

Note *p<0.05.

Furthermore, the results for reliability, convergent validity and discriminant validity have been provided in Table 6. According to [65–68], average variance extracted (AVE) values greater than 0.40 are acceptable if the composite reliability (CR) is greater than 0.70. Whereas, maximum shared values (MSV) and MaxR(H) values must be less than 1 [69]. The values of AVE, MSV, and MaxR(H) were all within the acceptable range. Since the square root of AVE of each latent construct (diagonal elements) is larger than all its correlations with other latent constructs, discriminant validity has been established [65].

**Table 6:** Validity analysis (convergent & discriminant) (N = 268).

Variables	AVE	Composite Reliability	MSV	MaxR(H)	1	2	3	4
**SOPEP**	0.502	0.916	0.375	0.927	**0.709**			
**SOPEA**	0.468	0.901	0.180	0.936	0.363	**0.684**		
**SOCNE**	0.643	0.926	0.375	0.935	0.613	0.352	**0.802**	
**SOSEI**	0.766	0.907	0.180	0.908	0.318	0.424	0.311	**0.875**

## Discussion

In order to bring together different views, shreds of evidence and facts both from scholars and practitioners about mores of the customer base for ecotourism industry, a rigorous procedure for scale development based on grounded theory, as suggested by [31], has been adopted. The final interpretation of the information is in the form of a new scale, which carries 4 dimensions comprising a pool of 32 items. It was then empirically tested for its validation of data collected from 268 Malaysian tourists.

The dimension ‘sense of obligation to care for the natural environment’ examines the pro-environment actions pertinent to the safety and protection of natural resources (i.e., air, water and land), reduction of the impacts on global warming and climate change, and development of eco-friendly behavior. This dimension has been extensively supported in the contemporary literature, and is identified as an intrinsic characteristic which can motivate an individual to oblige toward performing pro-environmental actions [6,8,39,41,42]. According to [6,8,39], people oblige themselves to save and protect natural resources, because they are limited. [6,8,41,42] suggest that the development of a sense of obligation to care for the natural environment actually motivates individuals to reduce their impacts on global warming and climate change, and develop an eco-friendly behavior regardless of what others think about them.

The ‘sense of obligation to practice eco-friendly activities’ was identified as another important intrinsic characteristic which can motivate an individual to oblige toward performing several pro-environmental actions such as traveling through environmentally sound modes of transportations (e.g., electric vehicles, hydrogen-fueled vehicles, compressed aired vehicles, Stirling-powered vehicles, and fine-tuned vehicles), practicing eco-friendly accommodation (or hotel) and recreation, reducing electricity usage, and increasing eco-friendly packaging usage. This dimension has also been substantially discussed in the existing literature (i.e., [6–16,21]). It is also worthwhile to note that developing a sense of obligation to practice eco-friendly activities motivates an individual to oblige toward traveling through environmentally sound modes of transportations even if it takes more time and/or is more expensive [7,9–16].

Likewise, the dimension ‘sense of obligation to purchase eco-friendly products’ seeks to appraise various pro-environmental actions such as purchasing recycled products, organic food items (e.g., fresh meat and dairy, processed foods, and frozen meals), organic clothing articles (e.g., outfit made of cotton, silk, jute, wool or ramie), and organic personal care products (e.g., made of plant or naturally occurring ingredients). This dimension has also been greatly endorsed by the current literature, for example, [16–20,43,44] suggest that sense of obligation to purchase eco-friendly products oblige people to purchase recycled and organic products.

Finally, a sense of obligation to support eco-friendly inventions (one of the dimensions) measures the pro-environmental action through supporting all those inventions (or innovations) which are less harmful to the environment (e.g., the electric airplane). This dimension has been perceived as an intrinsic characteristic by only a few researchers (such as [10,26,45]). For example, [26] noted that there exists a motivation among people to oblige toward traveling through the electric airplane (a likely eco-friendly invention soon).

## Conclusion, implications, and delimitations

Overall, this research has contributed to the body of knowledge in the hospitality and tourism management domain by introducing dimensions for ‘mores of the customer base’, which have not been identified in the previous research. This suggests that new research approaches may be required to incorporate the sense of obligation to care for the natural environment, support eco-friendly inventions, practice eco-friendly activities, and purchase eco-friendly products for making Pro-environmental actions.

In this study, a blend of qualitative and quantitative data has been used in a resourceful manner for the construction of a new scale of ‘mores of the customer base’. By using a bottom-up investigative approach, a breakthrough was made by advancing contemporary literature and adding value to the ecotourism industry. On the qualitative side, the major contribution of this study is the development of mutually agreed intended meanings and breadth of the theoretical concepts with the help of 17 scholars and 15 practitioners hailing from Asia, Europe and America. Also, the development of a measuring scale by adopting a rigorous procedure of scale development, as suggested by [31]. For example, the language experts were invited for a focus group session to address various areas, such as ambiguity, confusion, leadings, difficulties, skips, sensitivities and missing questions. On the quantitative side, the foremost contribution of this study is the refinement and validation of newly established scale through survey data collected from the 268 Malaysian tourists to provide a more worldwide view to the existing literature, because most of the existing literature in a similar context comes from western countries.

This new construct has achieved such a holistic level that has never been achieved in previous sub-optical scales. Therefore, a new pathway role of the refined scaled constructs can be investigated in future studies based on the assumption that the increased mores of the customer base is an actual source of inclination to buy ecotourism packages. Such confirmation is necessary, not only because a better understanding of the mores of the customer base will primarily support practitioners to gain high ecotourism market shares, but also to address the criticism that ‘tourism’ and ‘eco’ is an uncomfortable pair–with the increased popularity of an ecotourism destination, the limitations of environmental and cultural impacts on community become difficult–local community might not enjoy the benefits of an ecotourism destination due to increased crowd near to their residence, increased prices of items, and limited access to pasture land or water [70]. Besides, since this study emphasizes toward a holistic approach to achieve sustainable development, it would help accomplish two of the Sustainable Development Goals (SDGs) adopted by the General Assembly of the United Nations in their Agenda 2030 [32], namely, Sustainable Cities and Communities, and Responsible Consumption and Production.

This study is not without its limitations and future study directions. First, this new scale is only applied to measure mores of the customer base in usual circumstances. When it comes to facing a slightly unusual circumstance (e.g., expensive, time-taking, or social isolation, etc.), a more updated scale is required in future studies. For example, it has been a subject of discussion that an individual, believing to have an overall eco-friendly behavior, may change his own principles while hearing what others say (or do) about him [39]. Likewise, an individual deciding to travel through environmentally sound modes of transportations may change his decision if it turns out to be more expensive and/or time taking [7]. Likewise, an individual deciding to purchase organic products may change his decision if it turns out to be more expensive [16].

Second, the principle concept of this research restraints around studying the ‘sense of obligation’–a feeling (an emotional state or reaction) that something is the right thing to do. Since a ‘feeling’ may eventually end-up in the formation of a belief (an acceptance that something exists or s true), an extension of this new scale can be investigated, for example, by utilizing the concept of ‘environmental self-identity’–a belief that the environment is important to us and an important part of who we are’ [71].

Third, the data utilized in this study for refining and validation purposes were collected from tourists of one country (i.e., Malaysia), meaning that generalizability can potentially be limited (e.g., due to not considering perspectives of other cultures and nationalities). There could be other thoughts of lodging consumers who belong to diverse cultures/nationalities. Based on that, we may have conversations in study results, for example, consideration any of the constructs of this new scale (most probably the sense of obligation to care for the natural environment) as a high-order dimension. Therefore, it is recommended to consider cultural dissimilarities caused by lodging consumers of various ethnic groups (or nationalities) for future studies (or research models) to enhance the generalization of the outcomes.

Last, with the rapidly increasing growth of market segment applications in hospitality businesses, future studies are recommended to segment the population in terms of their unique consumer traits–such as gender, generational cohort, and single e-shopper versus group e-shoppers. A unique way to conduct a future study, for example, could be to measure mores of the customer base having similar personality traits (searching them through personality profiling algorithms used for social networking). In this way, the scale can be examined in terms of the diversified significance of each dimension among various segments.

## Supporting information

S1 Fig Confirmatory Factor Analysis (CFA) of the constructs. (DOCX)

S1 Data (RAR)
