# Supplementary figures and images for: Mores of the customer base for ecotourism industry: Development and validation of a new measurement scale
Source: PLoS One. 2021 Feb 18;16(2):e0246410. doi: 10.1371/journal.pone.0246410 (PMC7891728; doi:10.1371/journal.pone.0246410)

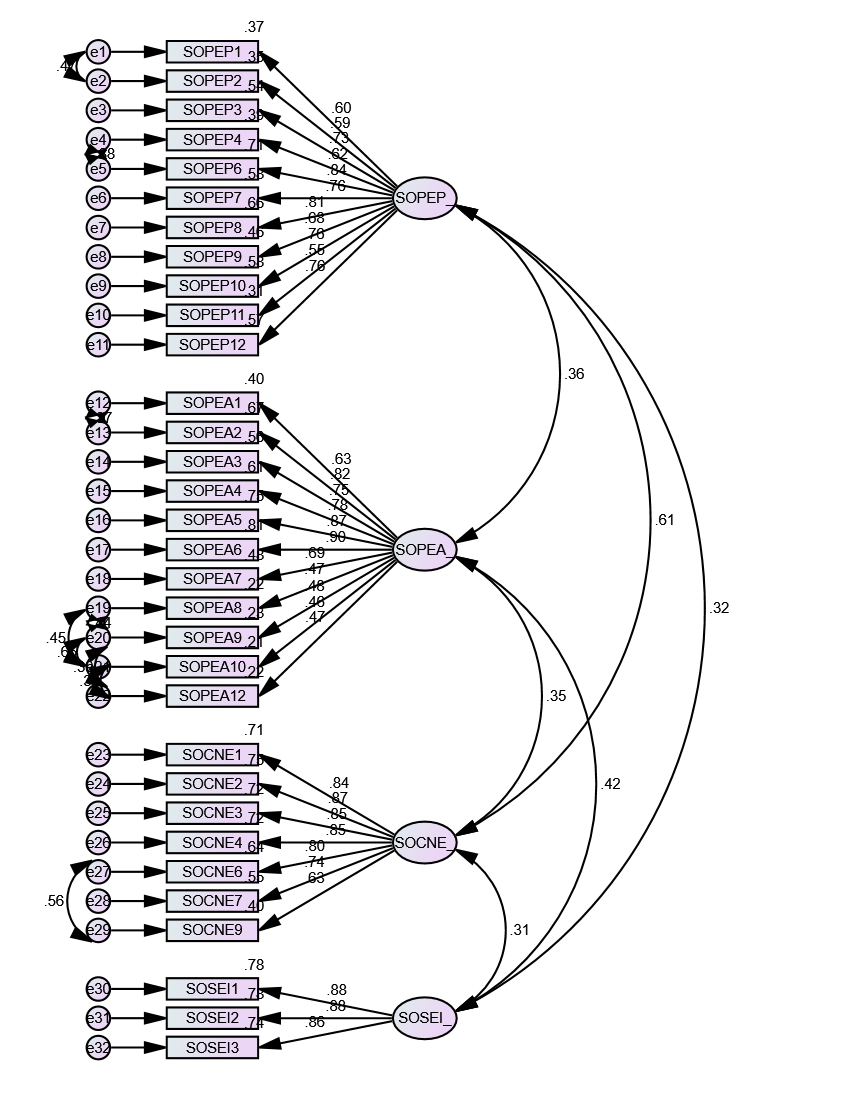

Supplement: S1 Fig — (DOCX) [file pone.0246410.s001.docx]
